# Microcarrier Screening and Evaluation for Dynamic Expansion of Human Periosteum-Derived Progenitor Cells in a Xenogeneic Free Medium

**DOI:** 10.3389/fbioe.2021.624890

**Published:** 2021-05-24

**Authors:** Kathleen Van Beylen, Ioannis Papantoniou, Jean-Marie Aerts

**Affiliations:** ^1^M3-BIORES: Measure, Model, and Manage Bioresponses, Division Animal and Human Health Engineering, Department of Biosystems, KU Leuven, Leuven, Belgium; ^2^Prometheus, Division of Skeletal Tissue Engineering, KU Leuven, Leuven, Belgium; ^3^Skeletal Biology and Engineering Research Centre, Leuven, Belgium; ^4^Foundation for Research and Technology – Hellas (FORTH), Institute of Chemical Engineering Sciences, Patras, Greece

**Keywords:** human periosteum-derived progenitor cells, microcarrier, star-plus, human platelet lysate, spinner flask cell culture

## Abstract

An increasing need toward a more efficient expansion of adherent progenitor cell types arises with the advancements of cell therapy. The use of a dynamic expansion instead of a static planar expansion could be one way to tackle the challenges of expanding adherent cells at a large scale. Microcarriers are often reported as a biomaterial for culturing cells in suspension. However, the type of microcarrier has an effect on the cell expansion. In order to find an efficient expansion process for a specific adherent progenitor cell type, it is important to investigate the effect of the type of microcarrier on the cell expansion. Human periosteum-derived progenitor cells are extensively used in skeletal tissue engineering for the regeneration of bone defects. Therefore, we evaluated the use of different microcarriers on human periosteum-derived progenitor cells. In order to assess the potency, identity and viability of these cells after being cultured in the spinner flasks, this study performed several *in vitro* and *in vivo* analyses. The novelty of this work lies in the combination of screening different microcarriers for human periosteum-derived progenitor cells with *in vivo* assessments of the cells’ potency using the microcarrier that was selected as the most promising one. The results showed that expanding human periosteum-derived progenitor cells in spinner flasks using xeno-free medium and Star-Plus microcarriers, does not affect the potency, identity or viability of the cells. The potency of the cells was assured with an *in vivo* evaluation, where bone formation was achieved. In summary, this expansion method has the potential to be used for large scale cell expansion with clinical relevance.

## Introduction

The rising amount of research toward cell therapies is translated in the increasing amount of registered clinical trials on ClinicalTrials.gov of which currently 1409 trials use adult mesenchymal stem/stromal cells (MSC) as a therapeutic cell source. Therapies using these cells target a wide range of diseases including bone disorders, cartilage damage or inflammatory diseases (Durand and Charbord, 2015). This work focusses on specific adult mesenchymal progenitor cells derived from the human periosteum due to their benefits in skeletal tissue engineering. This periosteum is a thin vascular membrane around most bones, situated between the cortical bone and the covering soft tissue and consists of an outer fibrous layer and an inner cambium layer containing adult mesenchymal progenitor cells (Allen et al., 2004). These human periosteum-derived cells (hPDCs) have similar characteristics as adult mesenchymal stromal cells (MSCs). Both cell types, MSCs and hPDCs, possess self-renewal capacity, express a specific set of MSC markers and are capable of differentiating into a variety of cell types, such as chondrocytes, osteoblasts and adipocytes, as well as myoblasts. The benefits of hPDCs are on the one hand the relatively easy accessibility and on the other hand their high bone regenerative potential (de Bari et al., 2006). They even have a higher growth and differentiation potential than bone marrow stromal/stem cells (BMSCs) (Duchamp de Lageneste et al., 2018). More specifically, hPDCs are valuable in skeletal tissue engineering for the regeneration of defects in long bones, as the periosteum is the main source of the cells involved in the callus formation during facture healing (Nilsson Hall et al., 2020). Treating critical size long bone defects using skeletal tissue engineering has the potential to repair large bone defects as well as joint surface defects. These critical defects were otherwise too large for the body to heal by itself and when left untreated, it could even result in the loss of a limb. Current treatments consist of bone void fillers, which can be natural, synthetic or a combination. However, the outcomes of these commercial bone void fillers remain unpredictable (Slevin et al., 2016).

In order to provide such cell therapies, there is a need to scale-up the expansion of cells since only a small fraction of the required amount of cells can be harvested from a single donor. A therapeutic dose of MSCs requires between 10^7^ and 10^9^ cells (Jung et al., 2012), while only 10^4^ to 10^5^ MSCs can be harvested from a single biopsy depending on the source of acquisition (Beitzel et al., 2013). Interesting strategies for large-scale expansion of MSC’s are investigated in the review of García-Fernández et al. (2020). Their article describes the importance of choosing the right bioprocess design, such as the culture medium formulation and addresses the different scale-up strategies (García-Fernández et al., 2020). Depending on the type of stem cell used during the therapy, different cell culture vessels are preferred (dos Santos et al., 2013). This work focussed on human periosteum-derived cells (hPDCs), which are an adherent progenitor cell type similar to MSCs. Adherent cells require a surface to attach to, which is typically the bottom of a flask, but could also be hollow fibers or small microcarriers in suspension. Since scale-up is essential to fulfill the demand for MSCs or other cell types used in regenerative medicine products (Olsen et al., 2018), it is important to select the most efficient production process toward large scale productions. MSCs and hPDCs have been expanded in multistack (Lambrechts et al., 2016b) and hollow fiber bioreactors (Jones et al., 2013; Nold et al., 2013; Rojewski et al., 2013; Hanley et al., 2014; Lambrechts et al., 2016a). However, certain challenges mostly related to sub-optimal cell harvest efficiency or lack of process flexibility have established suspension culture as an efficient and flexible set-up for adult progenitor cell expansion. Therefore, dynamic systems with microcarriers were investigated instead of the traditional planar culture systems thanks to the increased surface area to volume ratio.

The selection of an appropriate microcarrier for each cell type is crucial, since it influences the seeding efficiency, proliferation rate and harvest efficiency (Schnitzler et al., 2016). An overview of the most common used microcarriers in cell expansion is given in Table 1. Commercial microcarriers differ in core material, coating material, ionic surface charge, porosity, swelling upon hydration and size resulting in different seeding, proliferating or harvesting efficiency for a specific cell type. Important research into the full 3D morphologic characterization of microcarriers using a combination of microfocus X-ray computed tomography (microCT) and contrast-enhanced microCT (CE-CT) is recently reported by our group (de Bournonville et al., 2021).

**TABLE 1:** Manufacturing information of commercially available microcarriers, screened in this study.

Abbr.	Microcarrier	Manufacturer	Matrix	Coating	Hydration	Diameter (μm)	Surface area (cm^2^/g dry weight)
**Animal protein**							
Coll	Collagen	Sartorius	Cross-linked polystyrene	Porcine collagen		125–212	360
F3	FACT III	Sartorius	Cross-linked polystyrene	Porcine collagen cationic charge		125–212	360
CultiS	CultiSpher-S	Percell Biolytica AB	Macro porous cross-linked gelatin		Yes	130–380	
**Xeno-free**							
H2	Hillex II	Sartorius	Modified (cationic amine) polystyrene			160–200	515
Pl	Plastic	Sartorius	Cross-linked polystyrene			125–212	360
Pl+	Plastic-Plus	Sartorius	Cross-linked polystyrene	Cationic charge		125–212	360
St+	Star-Plus	Sartorius	Cross-linked polystyrene	Net positive charge		125–212	360
C1	Cytodex-1	GE Healthcare	Cross-linked dextran	positive charged DEAE groups	Yes	140–200	4400
C0	Untreated	Corning	Polystyrene			125–212	360
SY II	Synthemax II dissolvable	Corning	Cross-linked PGA polymer chains	Corning synthemax II	Yes	200–300	5000

A clear comparison of the expansion of MSC’s using the different microcarriers Cytodex-1, Star-Plus, Plastic, Plastic-Plus and HillexII is given in Loubière et al. (2019). Here, Star-Plus and Plastic-Plus are chosen as the best microcarriers for the cell culture of MSC’s derived from umbilical cord based on the criteria of cell attachment, expansion and detachment (Loubière et al., 2019). Health authorities are in favor of avoiding the use of animal components due to safety reasons and animal welfare (Cimino et al., 2017). Hence, the use of human platelet lysate (hPL) as a xeno-free alternative for fetal bovine serum (FBS) supplement has been suggested and evaluated in many studies (Xia et al., 2011; Gottipamula et al., 2012; Oikonomopoulos et al., 2015; Heathman et al., 2016). hPL is a xeno-free medium, containing platelet derived growth factor (PDGF), transforming growth factor beta 1 (TGF-b1), insulin like growth factor (IGF-1), and basic fibroblast growth factor (bFGF) (Gottipamula et al., 2012; Oikonomopoulos et al., 2015; Heathman et al., 2016). The abundance of these growth factors in combination with an environment more closely related to the physiological human body might explain the consistently higher proliferation and culture expansion rate of MSC in hPL media in comparison to FBS (Lohmann et al., 2012). In this study, we investigated the influence of commercial microcarrier types for the expansion of hPDCs in an hPL supplemented medium composition. We strived to verify expansion and harvest efficiency, while simultaneously evaluating the bone forming capacity of the dynamically expanded cells.

The goal of this work was to select the appropriate microcarrier for scalable expansion of hPDCs in a xeno-free medium. In order to do this, different commercial microcarriers were screened based on standard characteristics of seeding, proliferation and harvesting efficiency. Most microcarrier research uses *in vitro* techniques to assess the quality of the cells after expansion. However, this is not a guarantee for *in vivo* success (Nilsson Hall et al., 2020), especially when evaluating progenitor cells derived from different sources. Therefore, this work also evaluated the functionality of the dynamically expanded hPDCs grown on Star-Plus microcarriers by subcutaneous implantations in nude mice.

## Materials and Methods

### Experimental Set-Up

**FIGURE 1:**
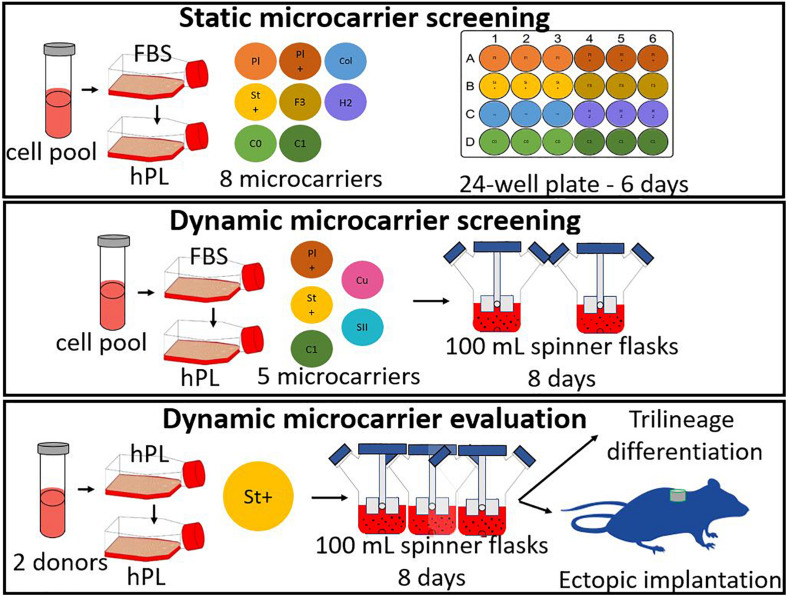
General scheme of the experiments, starting with a static microcarrier screening of 8 microcarriers and followed by a dynamic microcarrier screening of 5 microcarriers. The final experiment evaluated the hPDCs, which were dynamically expanded on the chosen microcarrier Star-Plus, *in vitro* as well as *in vivo*.

Three different types of experiments were performed for the microcarrier screening of ten different commercial microcarriers, shown in Table 1. The three experiments are briefly described hereafter and in more detail in the following sections. A scheme of all three experiments is represented in Figure 1.

The first experiment consisted of a broad static screening in well plates using eight microcarriers (Plastic, Plastic-Plus, Star-Plus, FactIII, HillexII, Collagen; Cytodex-1, and Corning untreated). A cell pool was seeded for each of the microcarriers in six individual wells of a 24-well plate for the evaluation of seeding efficiency and proliferation rate. 24 h after seeding, three wells were sacrificed for measuring the DNA content of the cells attached to the beads and the DNA of the cells in the supernatant. After 6 days of cell culture, the three other wells of each type of microcarrier were sacrificed to measure the total content of DNA on the cells attached to the microcarriers.

The second experiment selected three microcarriers (Plastic-Plus, Star-Plus, Cytodex-1) from the static screening and added Cultispher-S and Synthemax II dissolvable to perform a dynamic microcarrier screening experiment. The same cell pool as previous screening experiment was used in combination with the five microcarriers in a dynamic expansion. Each microcarrier type was cultured in duplicates in spinner flaks of 100 mL for 8 days. The DNA and metabolites were sampled daily at the same time, before medium replacement.

The third and final experiment evaluated the dynamic expansion of cells from two different donors using one specific microcarrier, Star-Plus. The cells of these two donors were cultured in triplicate spinner flasks resulting in a total of six spinner flasks for the duration of 8 days. After the dynamic culture, the quality of the cells was extensively evaluated.

### Static Microcarrier Screening Experiment

#### Human Periosteum-Derived Progenitor Cells

Human periosteum-derived progenitor cells (hPDCs), used throughout this study, were obtained from periosteal biopsies acquired in the university hospital of KU Leuven at Pellenberg, Belgium (Eyckmans et al., 2010). All patients filled-in the informed consent form of the clinical study, which was approved by the KU Leuven medical ethics committee. The screening experiments were performed with pooled cells from five female donors between the age of 10 and 17. After isolation, the cells were cultured for multiple passages at a seeding density of 5500 cells/cm^2^ in high glucose Dulbecco’s Modified Eagle’s Medium (DMEM) containing 1% sodium pyruvate, supplemented with 1% antibiotic-antimyotic (AA) and 10% serum. The serum added at biopsy for the screening experiments was fetal bovine serum (FBS). After several passages, the cells were frozen in liquid nitrogen until the start of the experiment.

The experiment started with thawing cells and expanding them for two passages in tissue flasks at a seeding density of 5500 cells/cm^2^. The decision of taking pooled cells cultured in FBS from biopsy for both microcarrier screening experiments was a practical choice, due to availability of cells. However, hPL was preferred and therefore the cells, which were prior to being frozen cultured in FBS, were deprived from FBS and changed to hPL. During the first passage after thawing at the start of the experiment, the cells were subjected to a serum starvation protocol. This protocol started by culturing the cells in 10% FBS. At confluency, the cells were washed with PBS and the media was replaced with 0.1% FBS. After 24 h of serum deprivation, the cells were harvested and subcultured in 7.5% hPL for at least one passage before the start of an experiment to give the cells time to adapt to the different serum. The choice of using 7.5% of hPL in the media at the start of each experiment was based on previous experiments, indicating that it is economically the best ratio for clinical grade hPL, which is also suggested in literature (Xia et al., 2011; Gupta et al., 2019).

#### Preparation Culture Vessels and Microcarriers

The vessels used for the static microcarrier screening were CoStar ultra-low attachment 24-well plates (Corning). Each of the eight types of commercial microcarriers (Plastic, Plastic-Plus, Star-Plus, FactIII, HillexII, Collagen; Cytodex-1 and Corning untreated) were seeded in six wells. All microcarriers were weighed to achieve a ratio of 6 cm^2^/mL, which is equal to 6 cm^2^ for each well. After weighing, if required according to manufacturing instructions, the microcarriers were hydrated in MiliQ water or PBS, and sterilized through autoclaving. The water or PBS from the microcarriers was replaced with complete DMEM, consisting of DMEM+AA+7.5% hPL, and incubated in the wells in 1 mL, 24 h prior to use.

#### Seeding Protocol

Cells were seeded at a density of 5500 cells/cm^2^, resulting in 33,000 cells per well. The pooled cells were added to the well plates while manually stirring the microcarrier suspension, to ensure a homogeneous dispersion. After seeding, the well plates were kept static in the incubators. Besides the six wells seeded with cells for each microcarrier, three additional control wells were seeded with cells but without microcarriers.

#### Harvesting Protocol

Tissue flasks and well plates were harvested according to the standard protocol of washing with PBS and incubating with TrypLE for 10 min. Followed by removing the cells from the bottom or microcarriers by force, either tapping the sides of the flask or pipetting the medium in the well up and down. The cell suspension was transferred to a falcon tube and medium was added to neutralize the enzymes. To separate the cells from the microcarriers, a 60μm filter was used on top of the falcon tube. The cell suspension was centrifuged at 1300 rpm for 10 min or 160G (Hettich Universal 320/320R centrifuge).

#### Cell Quantification

Cell counts were performed using 0.25% trypan blue and a Bürker hemacytometer. DNA samples were collected by sacrificing three whole wells, once after 24 h and a second time at the end of the culture period of 6 days. The DNA samples were collected in Eppendorf’s and washed twice with PBS by centrifuging the samples for 5 min and removing the supernatant. After removing the excess PBS, RTL buffer with 1% β-mercaptoethanol was added. This mixture was vortexed for 15 s and stored in −80°C. The DNA content in the sample was measured by following the manufactures protocol of the qubit fluorometer where Quant-iT^TM^ dsDNA HS reagent, Quant-iT^TM^ dsDNA HS buffer and Quant-iT^TM^ standard were used.

To measure the seeding efficiency, the DNA of the supernatant as well as the DNA on the microcarriers was measured separately. The seeding efficiency was calculated by dividing the DNA on day 1 attached to the microcarriers by the total amount of DNA found in the well on day 1, as presented in equation 1. The fold increase of the expansion was calculated by dividing the DNA on day 6 by the DNA on day 1, as represented in equation 2.

(1) $$\frac{DNA_{beads}\left(day1\right)}{DNA_{beads}\left(day1\right)+DNA_{supernatant}\left(day1\right)}$$

(2) $$\frac{DNA_{beads}\left(day1\right)}{DNA_{beads}\left(day6\right)}$$

#### Live-Dead Cell Viability Assay on Microcarriers

A 0.5 mL homogenous sample of microcarriers was taken on day 6 of the well plate culture period. This sample was placed in a suspension well plate and stained with calcein AM an ethidium homodimer-1(Invitrogen). Live cells have intracellular esterase activity that convert cell-permeant calcein AM to fluorescent calcein. Dead cells have damaged membranes, to which ethidium can enter and bind to nucleic acids. After staining, the samples were visualized using an inverted fluorescence microscope (IX83, Olympus). The live cells appear as green, while dead cells appear as red during imaging.

#### Actin and Nucleus Staining

A whole 1 mL well containing cells on microcarriers was used for actin and nucleus visualization. The medium was removed from the settled microcarriers and washed with 0.5 mL PBS. After removal of the PBS, the cells were fixated using 0.5 mL of 4% paraformaldehyde (PFA powder diluted in PBS), which was pipetted up and down. The cells with microcarriers were incubated in this fixation solution for 1 h at room temperature, while occasionally stirred. After the incubation period, the PFA was removed and the samples were washed with 0.5 mL PBS. This PBS was then removed and replaced by 0.5 mL of 0.1 M Glycine. After an incubation period of 15 min at room temperature, the samples were washed with PBS and stored in PBS at 4°C until staining.

1 mL staining solution was prepared with 1 μL 4′,6-Diamidino-2-Phenylindole (DAPI, 2.5 mg/mL stock solution, Invitrogen), 4 μL Alexa Fluor 488 Phalloidin (Phalloidin, 200 U/mL stock solution, Life technologies), 20 μL Triton X_100_ and 975μL PBS. The PBS from the fixated samples was removed and 0.5 mL staining solution was mixed in the sample by pipetting up and down. The samples were covered with aluminum foil and incubated at room temperature on a shaker platform for 1 h. After the staining incubation period, the samples are washed with PBS twice and kept in 0.2 mL PBS. The samples were visualized using an inverted fluorescence microscope (IX83, Olympus) or a confocal laser scanning microscope (LSM 880, Zeiss). DAPI stains the nuclei and appears as blue on the imaging, while Phalloidin will stain actin as green.

### Dynamic Microcarrier Screening Experiment

#### Human Periosteum-Derived Progenitor Cells

The same hPDCs pool of five young female donors as previous static screening experiment was used during this dynamic screening experiment.

#### Preparation Culture Vessels and Microcarriers

The vessels used for the dynamic expansion were 100 mL spinner flasks (Bellco Glass Cat. Number 1965-00100) with a diameter of 65 mm, a height of 135 mm, a center neck of 70 mm and two side arms of 32 mm. Before use, the spinner flasks were coated with Sigmacote (Sigma-Aldrich) by pipetting up and down 25mL sigmacote over all inner surfaces of the spinner flasks. After a night of air drying in the hood, the coating was verified by visual inspection of perfectly formed water droplets on the coated surface. The coated spinner flasks were then sterilized through autoclaving.

All five microcarriers (Cytodex-1, Star-Plus, Plastic-Plus, Cultispher-S, SynthemaxII dissolvable) were weighed to achieve a ratio of 6 cm^2^/mL, which is equal to 480 cm^2^ for each spinner flask with a working volume of 80 mL. After weighing, if required according to manufacturing instructions, the microcarriers were hydrated in MiliQ water or PBS, and sterilized through autoclaving. The water or PBS from the microcarriers was replaced with pure human platelet lysate (hPL) and placed inside the spinner flasks and in the incubator set at 37°C, 5% CO_2_, and 95% relative humidity. 2 h before the inoculation, the pure hPL was replaced by complete medium (DMEM-C), consisting of DMEM+AA+7.5% hPL (Loubière et al., 2019).

#### Seeding Protocol

Cells were seeded at a density of 5500 cells/cm^2^, resulting in 2.64×10^6^ cells per spinner flask. The pooled cells were seeded while the spinner flasks were positioned on the magnetic plate at a stirring speed of 30 rpm with 20 mL of DMEM-C to ensure a homogeneous seeding. 5 min after seeding, the stirring speed was set to 0 rpm for 2 h after which 20 mL of DMEM-C was added. This 5 min ON and 2 h OFF protocol was repeated during 8 h. After these initial 8 h, the stirring speed was set at 30 rpm during the next 16 h (overnight). 24 h after seeding, the volume was topped-up from 40 to 80 mL and the stirring speed was increased from 30 to 50 rpm.

#### Harvesting Protocol

After 8 days of the expansion process, the cells were harvested inside the spinner flasks (Nienow et al., 2014; Rodrigues et al., 2019). The decision to end the culture period after 8 days was based on average hPDC cell growth data cultured in hPL and in tissue flask, unrelated to the type of microcarrier in order to compare all cell culture experiments over the same culture period. The stirring was stopped to let the microcarriers settle inside the spinner flasks, followed by removing as much medium as possible. The microcarriers were washed with PBS until most of the medium was washed out. As much PBS as possible was removed before adding TrypLE and incubating it for 15 min at a stirring speed of 50 rpm, using the manufacturing protocol. To separate the cells from the microcarriers, the suspension was filtered with a steriflip (60 μm). The filter was washed with medium, to get as much cells, which were stuck between the microcarriers, through the filter and to balance the enzymatic reaction of TrypLE. To remove the TrypLE and medium mixture, the cell suspension was centrifuged at 1300 rpm for 10 min.

#### Cell Quantification

Cell counts were performed using 0.25% trypan blue and a Bürker hemacytometer. The metabolites were sampled daily at the same time, before medium replacement. The glucose, lactate and lactate dehydrogenase (LDH) in the sampled medium were measured using the Cedex Bio Analyser (Roche).

The DNA samples were collected and measured according to the previously described methods. In order to translate the DNA value to cell numbers, an additional experiment was performed to achieve a standard curve with nine values of known cell numbers ranging from 0 to 55000 cells/mL. The DNA of each cell sample was measured according to the method described above, resulting in a relation between known cell number and measured DNA content as described in equation 3 (Supplementary Figure 1, *R*^2^ = 0.998). A similar validation on the same cell type has been done in a previous study by Chen et al. (2012).

(3) $$DNA\left(\frac{ng}{mL}\right)=0.01^{*}\frac{Cells}{mL}+0.1166$$

### Dynamic Microcarrier Evaluation Experiment

The final experiment evaluated the dynamic expansion of cells from two different donors using one specific microcarrier, Star-Plus. The cells of these two donors were cultured in triplicate spinner flasks resulting in a total of six spinner flasks for the duration of 8 days.

#### Human Periosteum-Derived Progenitor Cells

The dynamic evaluation experiment was not performed on a cell pool, but on two different male donors, where donor 1 was 22 years old at the time of biopsy and donor 2 was 37 years old. The serum added at biopsy also differs from previous experiments. For the static and dynamic screening experiment, the added serum was fetal bovine serum (FBS), while for the dynamic evaluation, it was human platelet lysate (hPL). Therefore, there was no need for a starvation protocol in this experiment.

#### Preparation Culture Vessels and Microcarriers

The six spinner flasks and the Star-Plus microcarriers used for the expansion of the two donors in triplicates were prepared as described above.

#### Seeding Protocol

Spinner flask 1, 2, and 3 were seeded with cells from donor 1 and spinner flasks 4, 5 and 6 with cells from donor 2. The cells were seeded at a density of 5500 cells/cm^2^ in 80 mL medium as described above.

#### Harvesting Protocol

The cells were harvested inside the spinner flasks, similar to the previous described method. The differences between the harvesting of the screening experiment were the increased stirring speed and incubation time. Instead of incubating for 15 min at a stirring speed of 50 rpm, the cells were incubated for 20 min at a stirring speed of 150 rpm. The stirring speed was increased for the last 5 s to 200 rpm to mimic the tapping on the side of standard tissue flaks, which breaks up all cell agglomerates and detaches the cells from the microcarriers. A suspension sample is visually inspected under a bright field microscope to ensure the detachment of the cells. The same separation method was used as previously described to separate the cells from the microcarriers.

### Cell Quantification

Cell counts were performed using 0.25% trypan blue and a Bürker hemacytometer. The glucose, lactate, ammonium and pyruvate concentrations in the sampled medium were measured using the Cedex Bio Analyser. The DNA samples were collected and measured as described above.

#### Live-Dead Cell Viability Assay on Microcarriers

A 0.5 mL homogenous sample of microcarriers was taken on day 1, day 4, and day 6 of the spinner flask culture, while stirring the spinner flasks, to use for live-dead visualization as previously described.

#### FACS Analysis

Cells from both spinner flasks, after a cell culture period of 8 days, and from confluent tissue flasks were harvested to assess the presence of typical MSC immunophenotypic cluster of differentiation (CD) markers as well as the lack of hematopoietic markers. Flow cytometry was therefore performed using the human antibodies CD73-APC, CD-90-FITC, CD105-PE, CD-14- PerCP, CD20- PerCP, CD34- PerCP and CD45-PerCP (Miltenyi Biotec). Dead cell exclusion was performed using a viability dye (Zombie Aqua, BioLegend). An initial antibody titration to avoid nonspecific antibody biding was achieved following the protocol of Hulspas (2010). The BD Canto II was used for the flow cytometry analysis together with the software BD FACSDiva.

The full compensation setup contained control samples, Fluorescence Minus One (FMO) samples, negative control, dead cell exclusion and the condition samples. The control samples were performed using compensation beads (UltraComp eBeads Affymetrix eBioscience), which were only stained for one of the antibody colors: FITC, PE, APC, or PerCP control. All other samples used half a million cells from tissue flasks each. The FMO samples were stained with all antibodies, except for one. The negative control contained cells without any antibody, and the dead cell exclusion sample was stained only with the viability dye. The cells for the dead cell exclusion contained 50% live and 50% dead cells, achieved by placing the cells 5 min on ice and 5 min in a 60°C water bath. The interested condition samples, either with cells from tissue flasks or spinner flasks, were stained with all antibodies. RNA extraction, cDNA synthesis, and quantitative PCR

The DNA of 1 million cells was sampled at day 0 (before seeding) and day 8 (after harvesting). The DNA sample was centrifuged, the medium was removed and 600μL of RTL buffer with 1% β-mercaptoethanol was added. This mixture was vortexed and stored in −80°C. The RNeasy Mini kit (qiagen) was used to extract the RNA, followed by using the NanoDrop ND-2000 to quantify the amount of RNA. Synthesizing the cDNA was performed using PrimeScript RT reagent kit, Perfect Real Time (TaKaRa). The final concentration of 5 ng/μL was stored at −20°C until further analysis. QPCR was performed using a 9μL of the mastermix of RNA free water, reverse primer, forward primer and Fast SYBR green (Thermo Fisher Scientific) and 1μL cDNA of the sample. All samples were processed using a rotor gene in duplicates as well as the control sample with RNA free water for each primer. The settings used were a hold of 2 min at 45°C, a second hold of 95°C for 30 s and cycles at 95°C for 3 s and 60°C for 20 s. The results were analyzed using relative quantification (2^–ΔΔ*Ct*^) where the fold change of a specific gene of each sample minus the endogenous control was compared to that specific gene on day 0 of the same donor minus the endogenous control.

#### *In vitro* Trilineage Differentiation

Human periosteum-derived progenitor cells can potentially differentiate *in vitro* into chondrocytes, adipocytes and osteoblasts. This differentiation was induced with specific differentiation media, while control samples only receive basal medium.

Chondrogenic differentiation started with seeding 400,000 cells in 20μL DMEM-C with 7,5% hPL in 24-well plates, after 2 h 0.5 mL medium was added. After 24 h, the medium was replaced by chondrogenic differentiation medium containing basal medium, consisting of low glucose DMEM supplemented (Life Technologies) with 1% antibiotic-antimycotic, and a mix of 1X ITS+ Premix (Corning), 100 nM dexamethasone (Sigma), 20 μM Y-27632 inhibitor (Axon medchem), 1 mM ascorbic acid-phosphate (Sigma), 40 μg/mL proline (Sigma), 10 ng/mL TGF β1 (peprotech), 100 ng/mL GDF5 (peprotech), 100 ng/mL BMP2 (peprotech), 0.1 ng/mL BMP6 (peprotech) and 0.2 ng/mL FGF2 (peprotech). This medium was replaced every 2 or 3 days during a culture period of 21 days. At the end of the culture period, the trilineage differentiation was evaluated using alcian blue staining.

For Adipogenic differentiation, a density of 10 000 cells/cm^2^ was seeded in a 24-well plate in 0.5 mL DMEM-C. After 24 h, the medium was replaced by adipogenic differentiation medium, where the basal medium consisted of αMEM (Life technologies) supplemented with 1% antibiotic-antimycotic and 10% hPL. The adipogenic differentiation medium also contained 1μM dexamethasone, 10 μg/ml human insulin (Sigma), 100 μM indomethacin (Sigma) and 25 μM 3-Isobutyl-1-methylcanthine (IBMX) (Sigma). This medium was replaced every 2 or 3 days during a culture period of 14 days. The evaluation after the differentiation period was performed using oil red o staining.

Osteogenic differentiation was performed with a seeding density of 4500 cells/cm2 in a 24 well plate and 0.5 mL DMEM-C. After 48 h, the medium was replaced with osteogenic differentiation medium, which contained basal medium with 100 nM dexamethasone, 50 μg/ml ascorbic acid-phosphate and 10 mM β-glycerolphosphate (Sigma). The basal medium was DMEM-C supplemented with 10% hPL. The culture period was 21 days and the medium was replaced every 2 or 3 days. The osteogenic differentiation was evaluated using alizarin red staining.

#### *In vivo* Ectopic Implantation and Analysis

After 8 days of cell culture expansion, 1⋅10^6^ cells of each spinner flask after harvesting was seeded on NuOss scaffolds in a volume of 30μL separately. In total, six scaffolds were seeded and left overnight in 12-well plates in DMEM-C for the cells to adhere before implantation the day after. The implantation was performed ectopically on the back of 8 weeks old nude mice (Jackson Laboratory). After 8 weeks, the scaffolds were explanted and fixated overnight in 4% PFA before being switched to PBS.

#### Nano Computed Tomography Scans

To evaluate the amount of mineralized tissue, the scaffolds were scanned after explantation, on a Phoenix NanoTom M (GE Measurement and control) system using the following scanning parameters: x-ray voltage of 60 kV, current of 170 μA, tube mode 0, filter 0.2 mm Al, target diamond/tungsten. The acquisition parameters used were: fast scans of 15 min with exposure time of 500 ms, averaging of 1, skip 0, detector calibration 2 points, an average voxel size of 2.72 μm and 1800 images.

#### Histology

Following the nanoCT scans, the scaffolds were decalcified 10 times with at least 24 h in between using EDTA, paraffin embedded, sectioned (5μm) and stained for histologic analysis. The first staining was hematoxylin and eosin (H&E) to visualize the general structure and location of cells in between the left-over scaffolds. The other staining used was Masson’s trichrome, which visualized the connective tissue from the cells.

### Statistical Analysis

Results are presented as mean ± standard deviation. All statistical analysis were performed with a 95% confidence level for a one or two-sample *t*-test using Matlab version 2018b. The one-sample *t*-test was used when comparing gene expression data to day zero, the null hypothesis. This null hypothesis states that the data has a normal distribution with mean equal to zero and an unknown variance. A two-sample *t*-test is used with a null hypothesis of the two data sets being independent random samples from normal distributions with equal means and equal but unknown variances. In this work, this was used to verify if the data of a specific cell characteristic is significantly different between two donors.

## Results and Discussion

Both microcarrier screening experiments explored together a total of ten different commercial microcarriers, shown in Table 1, based on the following criteria: (a) the seeding efficiency, (b) the proliferation efficiency, (c) the harvest efficiency, (d) the quality of the cells after expansion, and (e) if the microcarrier is made of xenogeneic free material.

### Static Microcarrier Screening Experiment

Each of the eight types of commercial microcarriers (Plastic, Plastic-Plus, Star-Plus, FactIII, HillexII, Collagen; Cytodex-1, and Corning untreated) was evaluated in a 24-well plate for seeding efficiency and proliferation rate. Although the objective is to use a xeno-free microcarrier, this selection does include animal protein containing microcarriers to evaluate the difference in performance between the desired xeno-free microcarriers and the animal protein containing microcarriers.

**FIGURE 2:**
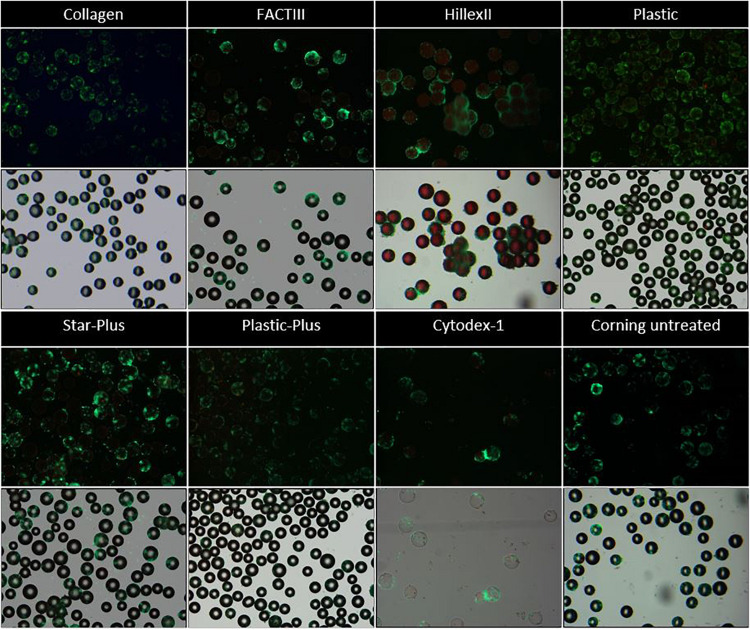
Live dead imaging of the microcarriers Collagen, FACTIII, HillexII, Plastic, Star-Plus, Plastic-Plus, Cytodex-1, and Corning untreated. The figures in row 1 and 3 represent the live-dead stainings, where live cells are stained green and dead cells are stained red. The figures in row 2 and 4 represent the merged images of bright field images combined with the live dead stainings to visualize the cells with respect to the microcarriers.

Using light microscopy, it was visible that the control wells without microcarriers contained agglomerates of cells after 3 days without the cells being attached to the bottom of the wells. This indicates that the ultra-low attachment coating of the well was sufficient to avoid competition with the microcarriers. Live-dead staining on the microcarriers showed that the cells attach to one or more microcarriers, leading to the formation of clumps as visualized in Figure 2, starting with a static microcarrier screening of 8 microcarriers and followed by a dynamic microcarrier screening of 5 microcarriers. The final experiment evaluated the hPDCs, which were dynamically expanded on the chosen microcarrier Star-Plus, in vitro as well as *in vivo*.

**FIGURE 3:**
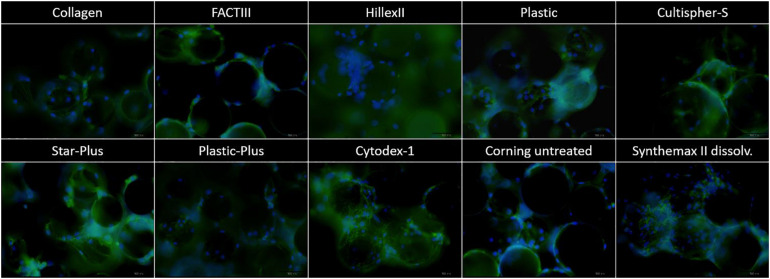
DAPI imaging of the microcarriers Collagen, FACTIII, HillexII, Plastic, Cultispher-S, Star-Plus, Plastic-Plus, Cytodex-1, Corning untreated, and Synthemax II dissolvable. The nucleus is stained blue, the actine is stained green and each picture has a width of 550 μm.

Figure 2 these live-dead stainings (starting with a static microcarrier screening of 8 microcarriers and followed by a dynamic microcarrier screening of 5 microcarriers. The final experiment evaluated the hPDCs, which were dynamically expanded on the chosen microcarrier Star-Plus, in vitro as well as in vivo. Figure 2 first and third row) are also merged with bright field imaging resulting in simultaneous live-dead as well as bright field imaging of the cells on the microcarriers (starting with a static microcarrier screening of 8 microcarriers and followed by a dynamic microcarrier screening of 5 microcarriers. The final experiment evaluated the hPDCs, which were dynamically expanded on the chosen microcarrier Star-Plus, in vitro as well as in vivo, Figure 2 second and fourth row). Additional DAPI stained samples of these eight microcarriers as well as the microcarriers Synthemax II dissolvable and Cultispher-S are shown in Figure 3.

In order to decide which microcarrier is best suited for a certain cell type, the interaction between the microcarrier and the cells could be studied. An important aspect in this regard is whether the cells spread homogenously over the microcarrier, or if they clump together and not use all the available surface area efficiently. Depending on the cell type, the morphological characteristics of the microcarriers could also increase the available surface area. For example, the Cultispher-S microcarriers are macro-porous and cells smaller than 10 μm could use the surface area inside the pores to adhere to de Bournonville et al. (2021). Another factor influencing the available surface area is the swelling factor of the microcarriers that require hydration, such as Cultispher-S, Cytodex-1 and Synthemax II dissolvable. Furthermore, the interaction between the cell and microcarrier will be influenced by the surface characteristics of the microcarrier, such as the coating material or ionic charge, which can be positively charged (Hillex II, Star-Plus, Plastic-Plus and FACT III, Cytodex-1), negatively charged (Synthemax II dissolvable) or neutral (Corning Untreated, Collagen, Cultispher-S and Plastic).

Future work could analyze the cell spreading objectively based on the amount of cells per microcarrier and the distance between the cells on each microcarrier, using 3D image analysis of a large dataset. However, initial observations in this work on cell spreading indicate that Hillex II performs worse than all other microcarriers based on the heterogenous distribution and the large clumping of cells and microcarriers. Counting the amount of nuclei on each microcarrier in the DAPI stained samples in Figure 3, results in a standard deviation of cells per microcarrier, which is biggest for Hillex II and smallest for microcarriers Cultispher-S, Star-Plus, and Plastic-Plus. Therefore it seems that these microcarriers allow a more homogenous spreading of the hPDCs, which is favorable in choosing the best microcarrier.

**FIGURE 4:**
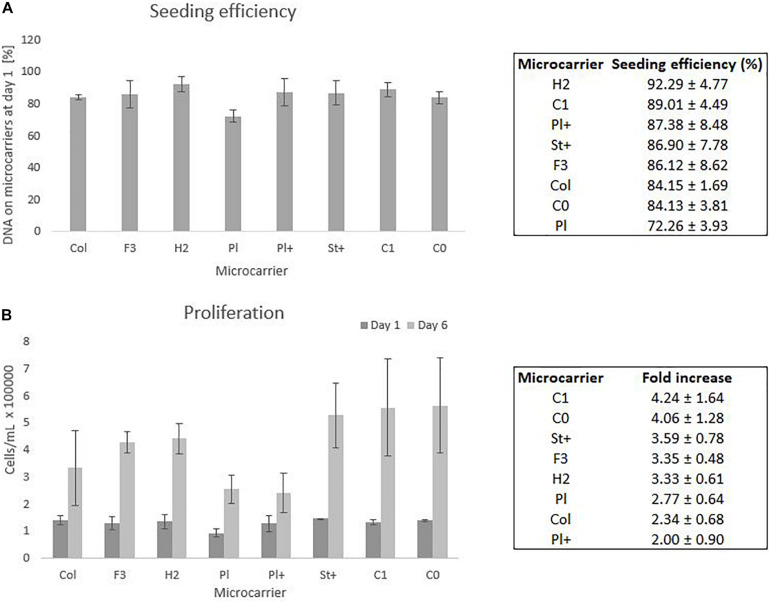
Static screening experiments of the microcarriers Collagen, FACTIII, HillexII, Plastic, Plastic-Plus, Star-Plus, Cytodex-1, Corning untreated. **(A)** The seeding efficiency is presented as the amount of DNA attached to the microcarriers after 24 h compared to the time of seeding. **(B)** The proliferation capacity of the cells is visualized as the amount of cells on day 1 compared to day 6. These cell counts are based on DNA calculations converted to cell counts.

From six wells of each condition, three whole wells were sacrificed on day 1 to measure all DNA present on the microcarriers as well as in the supernatant. The comparison between the DNA on the microcarriers and the DNA in the supernatant of day 1 gives an indication of the seeding efficiency as presented in Figure 4A. The other three wells were sacrificed after a cell culture period of 6 days to measure all DNA on the microcarriers. The DNA on the microcarriers of day 6 is compared to the DNA of day 1 to measure the proliferation of the cells on each type of microcarrier, which is shown in Figure 4B, where both DNA measures are converted to cell densities based on the relation explained by equation 3.

Statistical analysis of the seeding efficiencies with a significance level of 95% indicates several significant difference between microcarriers. The Plastic microcarrier has a significant lower seeding efficiency compared to HillexII, Cytodex-1, Corning untreated and collagen coated. In addition, HillexII has a significantly higher seeding efficiency compared to the collagen coated ones. Due to the high variations between the DNA on day 6 of the same microcarrier, there were no significant differences in the fold increase between the different microcarriers.

The three best performing microcarriers based on the average seeding efficiency are HillexII, Cytodex-1 and Plastic-Plus. For the average proliferation, the best performing microcarriers are Cytodex-1, Corning untreated and Star-Plus. Cytodex-1 is therefore the best overall performing microcarrier based on seeding efficiency and proliferation rate, which is in line with other work. A similar preference for Cytodex-1 compared to Hillex II, Plastic, Collagen and Plastic-Plus, was found by Schop et al. (2010) who focused on seeding efficiency as a selection criteria for the expansion of human bone marrow derived MSCs. Our study achieved a seeding efficiency in static culture of 89% (Figure 4) compared to a seeding efficiency of 57% by Schop et al. (2010), 80% by Frauenschuh et al. (2007), and 85% by Malda et al. (2003) (Malda et al., 2003; Frauenschuh et al., 2007; Schop et al., 2010).

This static screening is only an indication of how the cells perform on the microcarriers and is not representative for a dynamic cell culture expansion. Therefore this work used the most promising microcarriers based of this screening combined with information in literature to make a selection of microcarriers to be assessed in a dynamic environment.

HillexII was not selected due to the high amount of clumping, the heterogenous spreading of the cells and the fact that it absorbs phenol red from the DMEM-C media and is heavier than other microcarriers. Being heavier would mean that the speed of the impeller in a dynamic environment needs to be increased to assure suspension, which is not favorable for the cells due to shear stress (Betrachtungen, 1968). Gupta et al. (2018) described the effect of an initial culture period at a higher speed 60 rpm compared to 30 rpm, which resulted in a high accumulation of LDH. Therefore they hypothesized the relation of increase culture speed with cell death in the spinner flask system (Gupta et al., 2018). Plastic was also excluded for the following experiments due to the significant lower seeding efficiency compared to other microcarriers. FactII and Collagen did not perform significantly better than the xeno-free microcarriers, therefore we could exclude these animal protein containing microcarriers. The last microcarrier that was excluded for following experiments was Corning untreated, since preliminary results indicated difficulties in harvesting the cells. In addition, there was no specific coating attached to the microcarriers to facilitate the cells to attach to the microcarriers compared to Star-Plus and Plastic-Plus.

The final selection from these eight microcarriers that was assessed in a dynamic expansion are Cytodex-1, Star-Plus and Plastic-Plus.

### Dynamic Microcarrier Screening Experiment

The microcarrier screening experiment in a dynamic expansion used the three chosen microcarriers from the first static screening experiment (Star-Plus, Plastic-Plus, and Cytodex-1) and added two interesting extra microcarriers (CultiSpher-S and Synthemax II dissolvable), which are both dissolvable. These were not yet included in the first screening experiment due to availability. However, since they have such an interesting characteristic of being dissolvable, they would have been included in the dynamic screening experiment regardless of their static performance.

**FIGURE 5:**
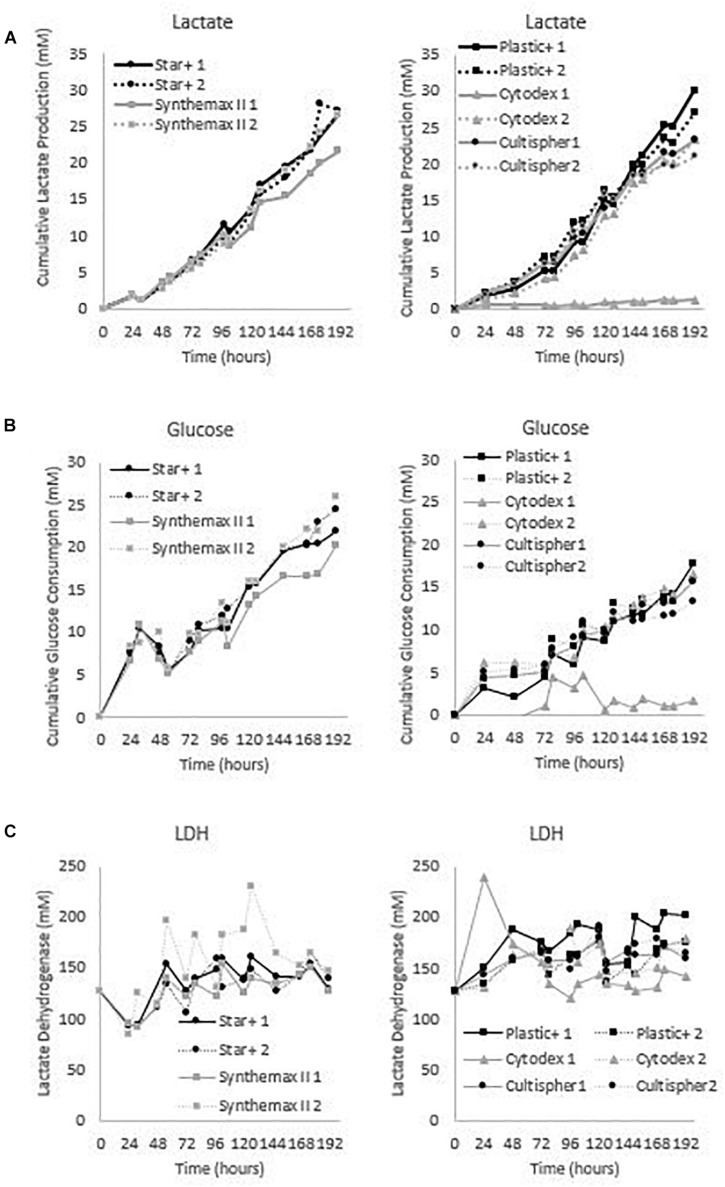
Metabolic profiles for the dynamic screening experiments of the microcarriers Star-Plus, SynthemaxII dissolvable, Plastic-Plus, Cytodex1, and Cultispher-S. The experiments were performed in duplicates for each of the microcarriers. **(A)** Cumulative lactate measurements, **(B)** cumulative glucose measurements and **(C)** Lactate Dehydrogenase (LDH) measurements.

These five microcarriers were all cultured in spinner flaks of 80 mL working volume in duplicates for 8 days. Although duplicates are not statistically valid data, there is still interesting information we can deduct from the metabolic results of the dynamic screening of the five microcarriers (Star-Plus, Plastic-Plus, Cytodex-1, CultiSpher-S and Synthemax II dissolvable), which are represented in Figure 5 and Table 3. What is immediately noticeable is the lack of cell growth in the second spinner flask with Cytodex-1 microcarriers. There was no lactate production, there was almost no glucose consumption and the LDH values spiked in the first metabolite sample after 24 h of cell culture. Therefore we will exclude the second spinner flask of Cytodex-1 in further discussions. Overall, the lowest glucose concentration was 13.12 mM and the highest lactate concentration was 17.35 mM indicating that there was no glucose limitation or lactate inhibition.

**TABLE 2:** Fold increase data for hPDCs expanded during dynamic screening experiment of 8 days.

Microcarrier	Fold increase before harvest	Fold increase after harvest
Plastic-Plus	7.5 ± 0.2	1.5 ± 1.1
Cytodex-1 (spinner flask 2)	8.0	0.09
Cultispher-S	3.3 ± 0.7	1.6 ± 0.4
Synthemax II dissolvable	4.9 ± 1.8	4.0 ± 1.4
Star-Plus	4.4 ± 0.4	1.6 ± 0.3

**TABLE 3:** Metabolites data for hPDCs expanded during dynamic screening experiment of 8 days.

Microcarrier	Spinner flask nr	Minimum Glucose (mM)	Maximum Lactate (mM)	Maximum LDH (U/L)
Plastic-Plus	1	15.48	17.35	202.9
	2	16.98	14.4	177.05
Cytodex-1	1	19.37	1.81	239.29
	2	16.39	13.87	190.32
Cultispher-S	1	17.34	13.79	180.49
	2	17.03	13.44	190.56
Synthemax II dissolvable	1	16.15	12.21	153.02
	2	13.12	14.93	229.55
Star-Plus	1	13.53	14.26	161.57
	2	14.43	14.63	165.24

The metabolic readouts collected in this experiment are used as a ‘surrogate marker’ for cell proliferation. The first indication of cell growth is a low minimum glucose concentration or high glucose consumption, since an increase in cells would require an increase in nutrient consumption. A second and similar cell growth indication is based on lactate, where an increase in cells would result in an increase of waste products (Schop et al., 2009). And finally, high LDH concentration are used to indicate cell death (Lobner, 2000). Based on these assumptions, both spinner flaks with Star-Plus microcarriers have the best metabolic profiles in relation to cell growth. They combine a high glucose consumption, intermediate lactate production and low LDH values. On the other hand, both Cultispher-S spinner flasks indicate a low amount of cell proliferation based on the lowest glucose consumption, rather low lactate production and rather high LDH concentrations. Cytodex-1 is similar to Cultispher-S but performs slightly better based on the metabolite indications. Both Plastic-Plus and SynthemaxII have contradicting metabolic profiles. Plastic-Plus (1) and SynthemaxII (2) have high values for glucose consumption and lactate production but also a high LDH value, which might indicate a high cell growth combined with a high cell death. For Plastic-Plus (2) and SynthemaxII (1), the glucose consumption and lactate production are lower as well as the LDH values indicating a slower cell growth with lower cell death.

The cell quantity of the experiment was only measured at seeding and at harvesting since intermediate sampling of only 250μL was not representative for the whole spinner flask due to heterogenous sampling. In an effort to measure the cell growth during cell culture, daily DNA samples were taken but there was too much clumping, especially for Cultispher-S, to be representative for the whole spinner flask. Increasing the sample volume would make the sample more representative, but it would also influence the cell growth number. An alternative would be to harvest whole spinner flasks every day. However, the metabolites data described above gives an indication of cell growth or cell death through the cell culture period.

**FIGURE 6:**
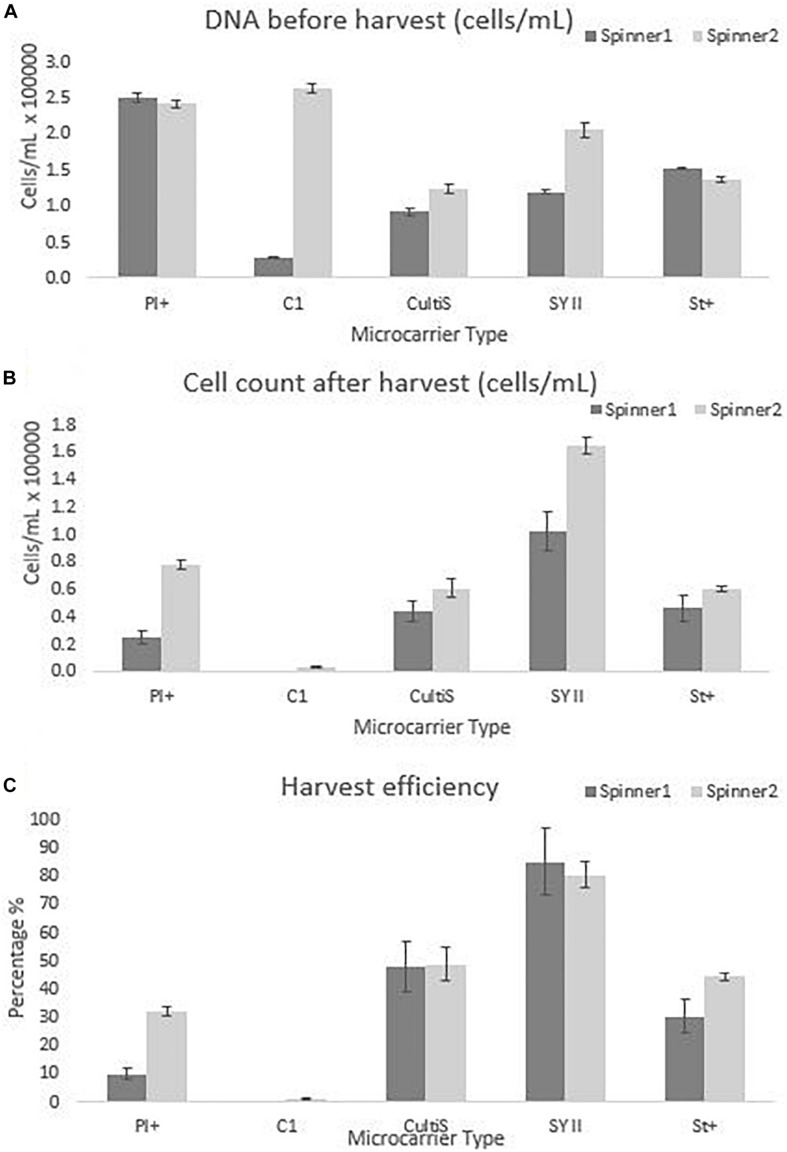
Cell quantity observations for the dynamic screening experiments of the microcarriers Star-Plus, SynthemaxII dissolvable, Plastic-Plus, Cytodex1, and Cultispher-S. The experiments were performed in duplicates for each of the microcarriers. **(A)** The DNA amount of the cells attached to the microcarriers on day 8 before harvest was measured and converted to a cell quantity. **(B)** The cells after harvest were measured for each spinner flask and **(C)** the harvest efficiency was calculated based on the comparison of the cells before harvest with the cells after harvest.

The cell number estimate before harvest was performed by sampling 4 mL of the cell culture and measuring the DNA content. This DNA content was translated to cell number using equation 3, the result is visualized in Figure 6 and Table 2. However, the harvesting protocol used to obtain these results was the one suggested by the manufacturer. These experiments were performed before investigating more optimal harvesting protocols, since optimizing the harvesting of all possible microcarriers was not in the scope of this project. Once a microcarrier was chosen, it was valuable to investigate the harvesting protocol of that specific microcarrier. Ideally this screening experiment could be repeated in case the harvesting protocol of all microcarriers is optimized. Using the manufacturing protocol means that the cells were harvested using a low stirring speed of 50 rpm for 15 min instead of the optimized protocol used in final dynamic microcarrier evaluation experiment, where the stirring speed is increasing toward 150 rpm with an additional 5 s at 200 rpm.

The dynamic microcarrier screening experiment in this work shows proliferation results for hPDCs seeded at 33000 cells/mL and cultured for 8 days to reach a fold increase before harvesting of 8.0 for Cytodex-1, 7.5 ± 0.2 for Plastic-Plus, 4.9 ± 1.8 for Synthemax II dissolvable, 4.4 ± 0.4 for Star-Plus and 3.3 ± 0.7 for Cultispher-S (Table 2). Data from Gupta et al. (2018), also working on the expansion of hPDC in spinner flasks, presented a fold increase of 3.2 ± 0.64 after 12 days of hPDCs expansion on Cultispher-S in Fetal Bovine Serum (FBS) based medium (Gupta et al., 2018). However, it is important to be able to harvest the cells from the microcarriers to be used in cell therapies. Since the harvesting in the dynamic microcarrier screening experiment was performed using the manufacturer’s protocol without optimization, this was rather low. The final fold increase of the cells after harvesting was therefore almost zero for Cytodex-1, 1.5 ± 1.1 for Plastic-Plus and CultiSpher-S, 1.6 0.3 ± for Star-Plus and 4.0 ± 1.4 for Synthemax II dissolvable (Table 2).

From the results of both static and dynamic screening, we selected Star-Plus as an interesting xeno-free microcarrier for the expansion of hPDC to be evaluated in the third experiment. Synthemax II dissolvable was initially the preferred microcarrier for the follow-up experiment due to the simplicity of harvesting, but the microcarrier was discontinued and could not be re-ordered at that time. Similar preferences for Synthemax II dissolvable were reported by Rodrigues et al. (2019), who investigated the expansion of human induced pluripotent stem cells (hiPSCs) on microcarriers. Synthemax II dissolvable resulted in a fold expansion of 4.0 ± 0.8 after 5 days of cell culture (Rodrigues et al., 2019). The reason for not selecting Cytodex-1, was due to the extreme low harvest efficiency, which was also reported in previous research. For example, Kehoe et al. (2012) published harvest efficiency data of 12% for Cytodex-1, whereas we only recovered 1%. Loubière et al. (2019) presents similar findings for the expansion of umbilical cord derived wharton’s jelly MSCs, where cells remain attached to Cytodex-1 and Star-Plus and Plastic-Plus are the preferred microcarriers. Plastic-Plus is also preferred in the work of Petry et al. (2016) where fold expansions of 16.4 and 13.8 are achieved after 7 and 6 days of culturing human Umbilical Cord MSCs (Petry et al., 2016). However, they did not compare with Star-Plus, probably because Star-Plus is the newest microcarrier of the SoloHill microcarriers (Sartorius), released at the end of 2015.

### Quality Assessment After Star-Plus Expansion

After the initial screening experiments, this work investigated the potential of Star-Plus microcarriers for the expansion of hPDCs. Six different spinner flasks were used, where two donors were expanded in triplicates for 8 days. The quality of the cells after cell expansion on Star-Plus microcarriers was assessed. Donor 1 was 22 years old during biopsy, while donor 2 was 37 years old. Based on literature, we suspect that this age difference could cause differences between the donors regarding their *in vitro* and *in vivo* potential (De Bari et al., 2001). After 8 days of cell culture, the cells were harvested using an improved harvesting method compared to the screening experiments, where the manufacturer’s protocol was followed. This improved protocol was based on the work of Nienow et al. (2014), where the cells were incubated for more than 15 min in an enzymatic detachment solution while being stirred at 150 rpm instead of the suggested 40 rpm of the manufacturer, with an additional 5 s at 200 rpm. Their work increased the harvest efficiency of Plastic microcarriers from less than 5 % to higher than 95% (Nienow et al., 2014). In our work, the harvesting efficiency for Star-Plus increased from 37% in the dynamic screening experiment up to 97% in dynamic evaluation experiment. This method was also used in the microcarrier screening work of Rafiq et al. (2016).

**FIGURE 7:**
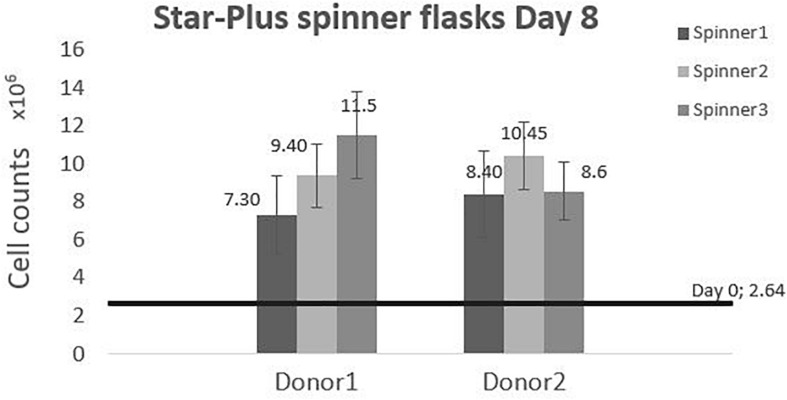
Cell counts of the dynamic Star-Plus evaluation for each of the triplicate spinners of each donor.

The cells harvested after 8 days of cell culture in spinner flasks are visualized in Figure 7. The average final cell number, cell density and fold increase after harvesting is shown in Table 4. This fold increase of 3.56 for donor 1 is similar to the 2D control of the tissue flask expansion previous to the start of the spinner flask expansion. When extrapolation the cell counts after a 5 day expansion in the tissue flask to 8 days, the fold increase would be 3.22. Due to the high variations in cells harvested from donor 1, there is no statistical difference between the two donors. This variation could be caused by the order of harvesting, where donor 1, spinner flask 1 was harvested first, followed by spinner flask 2 and 3, after which donor 2 was harvested in the same sequence. Increasing knowledge about harvesting the cells throughout the process could result in increased numbers of cells harvested according to the sequence of the three spinner flasks. Besides cell counts, the following evaluations indicate a visible difference between donor 1 and 2. However, cells from both donors expanded on Star-Plus retain their chondrogenic potential, *in vitro* as well as *in vivo*.

**TABLE 4:** Cell quantity after harvesting the cells cultured for 8 days in 6 different spinner flasks.

	Donor 1	Donor 2
Cell counts	9.40E+06 ± 2.10E+06	9.13E+06 ± 1.14E+06
Cell density	19 583 ± 4 375	19 028 ± 2 381
Fold increase	3.56 ± 0.80	3.46 ± 0.43

*Two donors each cultured in triplicates, starting at 2.46⋅× 10^6^ cells and 5500 cells⋅cm^–2^.*

**FIGURE 8:**
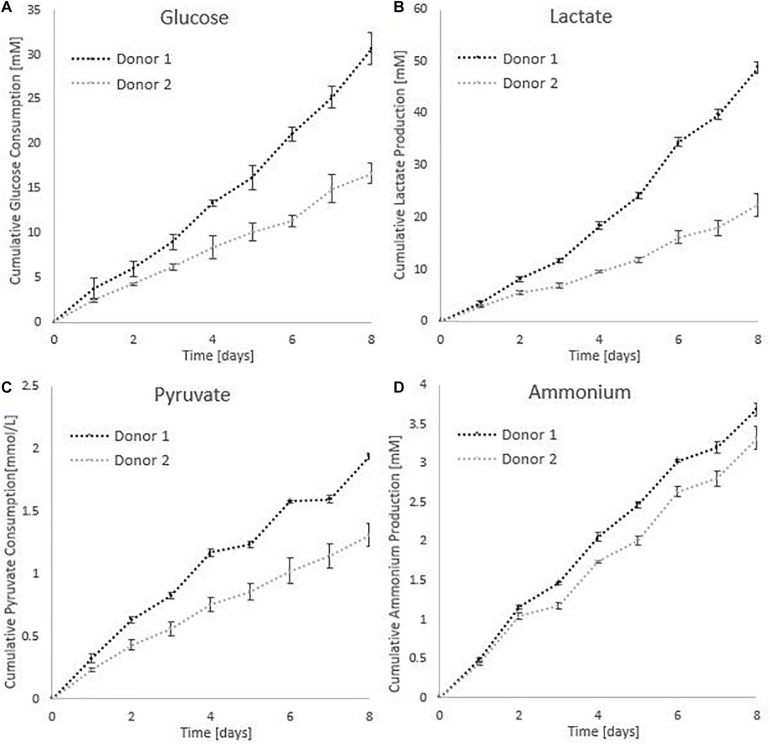
Metabolic profiles for the dynamic Star-Plus evaluation for an average of the triplicate spinners for each of the donors. **(A)** Cumulative glucose measurements, **(B)** cumulative lactate measurements, **(C)** pyruvate measurements, and **(D)** ammonium measurements.

All cumulative metabolite concentrations were significantly different on day 8 between donor 1 and donor 2, using a two-sample *t*-test with 95% confidence level. The metabolic activity of the cells is visualized in Figure 8. According to the findings of Schop et al. (2009), growth inhibition for human MSC’s occurs at 35.4mM for lactate concentrations and 2.4 mM for ammonia (Schop et al., 2009). The medium in the spinner flasks of donor 1 reached a maximum lactate level of 25.39 mM at day 7, while the spinner flasks of donor 2 reached a maximum lactate level of 11.84 mM at day 7. Highest ammonium levels for donor 1 were reached on day 5 with a value of 1.75 mM, whereas the culture medium reached a maximum of 1.61 mM at day 7. Due to the high glucose concentration of 25 mM in the medium, the concentration never dropped below 10 mM during the whole cell culture expansion. In addition, the pyruvate concentration starting at 0.95 mM never drops below 0.20 mM. Therefore we can assume there were no inhibitory effects due to glucose or pyruvate depletion or due to a high accumulation of lactate or ammonia.

**FIGURE 9:**
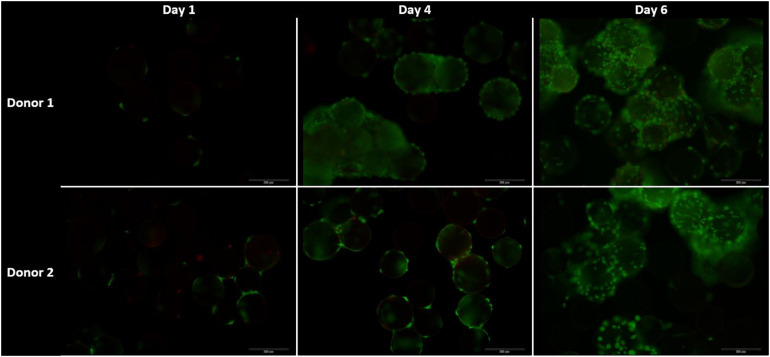
Live dead stainings for the dynamic Star-Plus evaluation on day 1, 4, and 8 for both donor 1 and donor 2. Live cells are stained green by calcein and dead cells are stained red by ethidium.

The live-dead staining of the cells on day 1, 4, and 6 of the cells on microcarriers are shown in Figure 9. On day 4, the amount of cells per microcarrier from donor 2 were visibly lower than the density of cells per microcarrier from donor 1. In addition, agglomeration is visible early on in the cell culture expansion, which could limit the cell growth. One method to decrease the agglomeration size is to add more microcarriers to increase the surface area (Ferrari et al., 2012). Another method is to increase the impeller speed in order to reduce the agglomerate size (Jossen et al., 2018). However, increasing the speed of the impeller should be properly investigated, because it could cause unwanted shear stress on the cells (Hewitt et al., 2011).

**FIGURE 10:**
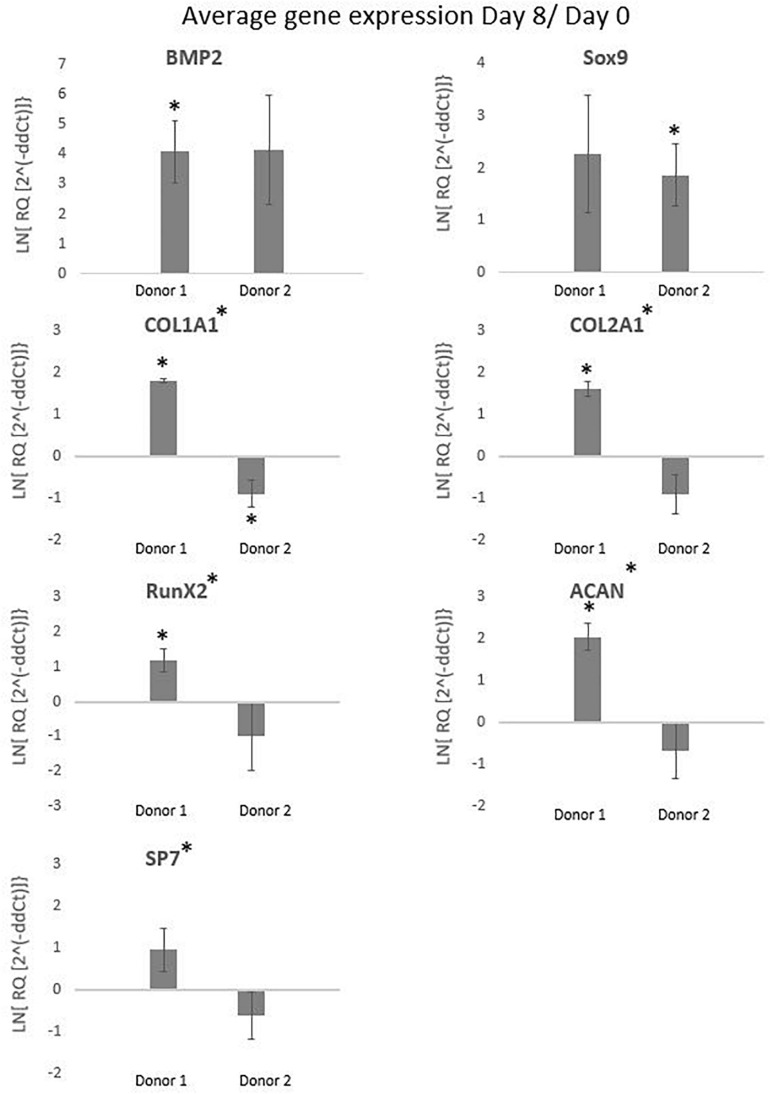
The graphs represent the average gene expressions from hPDCs cultured on Star-Plus in triplicate spinner flasks for two different donors after 8 days compared to day 0. The analyzed genes were bone morphogenetic protein 2 (BMP2), collagen I (COL1A1), RUNX family transcription factor 2 (Runx2), osterix (SP7), 573 sex determining region box 9 (Sox9), collagen 2 (COL2A1), and aggrecan (ACAN). The test uses a significance level of 95%, where (*) placed besides the gene name indicates a significant difference between donor 1 and 2 and (*) placed at the value of a donor indicates the significant difference between that donor on day 8 compared to day 0.

The International Society for Cellular Therapy (ISCT) proposes as a minimal criteria for MSCs that the markers CD73, CD90, and CD105 should be higher than 95% and the hematopoietic markers (CD14, CD20, CD34, and CD45) should be lower than 2% (Dominici et al., 2006). The FACS results for the cells cultured in spinner flaks compared to tissue flasks are represented in the Supplementary Figure 2, where the average expression of CD73 and CD90 are both higher than 95% and the hematopoietic markers are lower than 2%, while the expression of CD105 is reduced to an average of 90%. However, the reduced expression of CD105 has been observed in other literature using dynamic 3D cultures and is reported to be reversible when replating on 2D plastic tissue culture. The causes suggested in literature for the decrease are a too long detachment period, high concentrations of harvesting agent or high agitation rates (Brown et al., 2007; Potapova et al., 2008; Frith et al., 2010; dos Santos et al., 2011). Similar results for both a decrease in CD90 and CD105 were observed by Gupta et al. (2018, 2019) after the expansion of hPDCs on Cultispher-S in spinner flasks, which were described as non-significant. To conclude from these results, the hPDCs immunophenotypic markers were not permanently altered due to the spinner flask culture period and the following *in vivo* experiments will give a better inside in the impact of spinner flasks on the potency of the cells. The gene expressions visualized in Figure 10 are averaged over the three spinner flasks for each donor. These gene expressions on day 8 of the cell culture are compared to day 0 for bone morphogenetic protein 2 (BMP2), collagen I (COL1A1), RUNX family transcription factor 2 (Runx2), osterix (SP7), sex determining region box 9 (Sox9), collagen 2 (COL2A1), and aggrecan (ACAN). The significant difference between each donor with the control on day 0 or the difference between the donors is calculated using the means and standard deviations of the biological triplicates and indicated by the symbol (^∗^). In case a donor is significantly different from 0, the symbol is presented above the graph of that donor and in case the donors are significantly different from each other, the symbol is indicated besides the graph title from that gene. These results indicate that none of all the genes are simultaneously significantly different for both donors in the same direction. What is visible, is that for Col1a1 both donors are significantly different from the control on day 0, but for donor 1 this is a significant increase, while for donor 2 this is a significant decrease. The effect on gene expression of using Star-Plus in the expansion of the hPDCs is inconsistent between the two donors, which suggests that the influence is more related to donor variability than the expansion on microcarriers.

Sox9, a transcription factor indicative of chondroprogenitor cells, is upregulated for donor 2 and the potent bone inducer BMP2 for donor 1, indicative for genes related to differentiation via the osteogenic lineage (Ikeda et al., 2005; Ji et al., 2017). Although both Sox9 and BMP2 show a distinct upregulated trend, the fold change in both cases is rather low. The down regulation of collagen related cells in the case of donor 2 might suggest that cells are still in a proliferative state and have not yet reached confluency, which would allow secretion of collagen-based extracellular matrix (Tucker et al., 2020). This discrepancy in potency between donors is an integral challenge of the autologous cell therapy field but the qualitative match between *in vitro* measured quality attributes and subtle *in vivo* differences provides hope that markers for evaluating this difference could be develop in the future.

**FIGURE 11:**
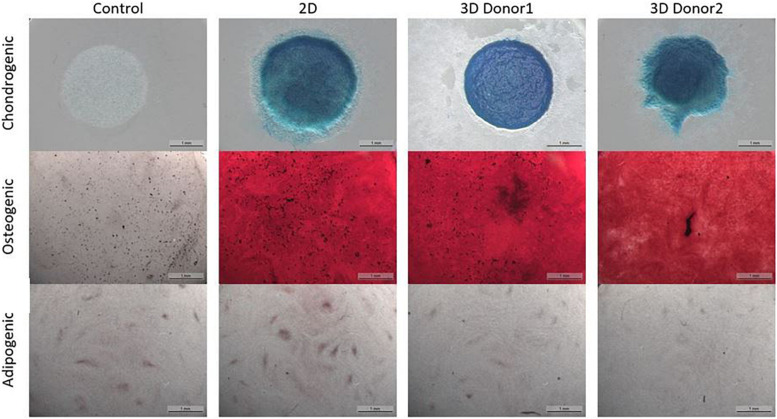
Trilineage differentiation of the dynamic Star-Plus evaluation for a control sample without differentiation medium, a 2D sample from cells cultured in tissue flasks and 3D samples from cells of two different donors cultured in spinner flasks on Star-Plus microcarriers.

The chondrogenic, osteogenic and adipogenic differentiation after 2 weeks or 3 weeks of differentiation for the cells culture during 8 days in spinner flasks are visualized in Figure 11. The trilineage differentiation after expansion in spinner flasks of both donors compared to the control of a 2D expansion on tissue flasks, confirm that the cells retain their initial bone and cartilage forming potency.

**FIGURE 12:**
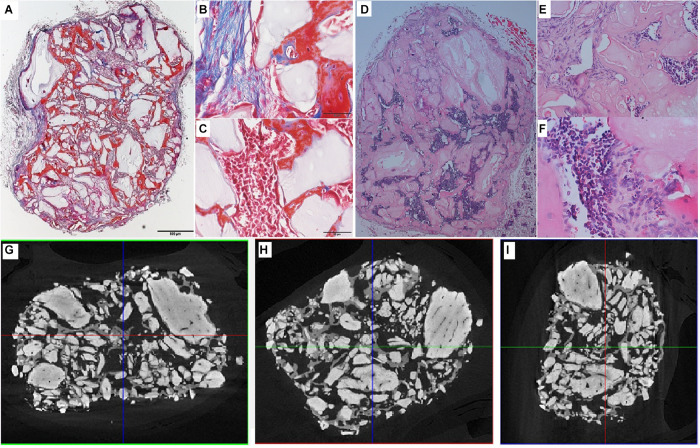
Visualization of explant donor1 spinner flask1 results. **(A–C)** Masson’s Trichrome staining. **(D–F)** H&E staining. **(G–I)** nanoCT scans 2D sections in all 3 planes.

**FIGURE 13:**
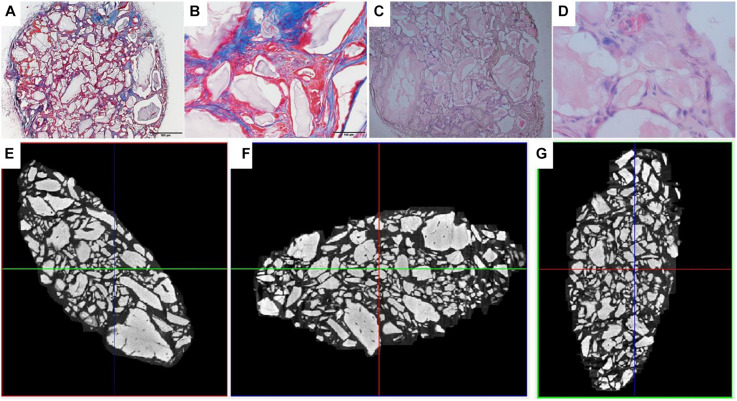
Visualization of explant donor 2 spinner flask1 results. **(A,B)** Masson’s Trichrome staining. **(C,D)** H&E staining. **(E–G)** nanoCT scans 2D sections in all 3 planes.

Analysis of the CT scans from the scaffolds, seeded with cells after 8 days of spinner flask expansion and after 8 weeks of implantation in nude mice are shown in Table 5. The same table also represents the control scaffolds, which were implanted with cells cultured statically in 2D tissue flasks. These results show that no matter what culture system is used, there is a significant difference between donor 1 and 2 when looking at the bone formation. What is also important to notice is that there is no significant difference in bone formation between the two culture systems. This suggests that the cells cultured in spinner flasks with Star-Plus microcarriers achieve similar bone volume formation as the cells cultured in the standard used tissue flasks. In addition, donor 1 has a significant higher bone formation compared to donor 2, both after the 2D as well as 3D expansion. For donor 1, a more developed bone domain has been developed within the CaP scaffolds with a clear presence of bone marrow compartment, suggesting a more rapid process of bone tissue formation. This same trend is visible in the hematoxylin-eosin (H&E) as well as Masson’s trichrome stainings, which are presented together with the CT scans in Figure 12 for donor 1 and Figure 13 for donor 2.

**TABLE 5:** Bone percentages analyzed from CT scans of cells implanted on NuOss scaffolds after 8 days of expansion on spinner flasks compared to cells only expanded in tissue flasks.

	Spinner flask bone volume (%)	Tissue flask bone volume (%)
Donor 1	9.80 ± 3.34	8.96 ± 0.72
Donor 2	3.85 ± 0.76	2.80 ± 1.77

Besides these *in vitro* methods, which are mainly used in other research on microcarriers, this work also uses *in vivo* bone forming experiments to assess the potency of the cells. The same conclusion for *in vivo* as the *in vitro* methods can be made, namely that both cell batches after the spinner flask expansion of the two different donors have no significant difference in bone formation compared to their 2D control.

Such *in vivo* bone forming experiments are used in other research, also to assess the quality of cells after a cell expansion process under specific conditions (Weiss et al., 2012). The work of Gupta et al. (2019) used this type of experiments to validate the positive effect of hPL on the *in vivo* bone forming potential of hPDCs expanded on Cultispher-S microcarriers in spinner flaks (Gupta et al., 2019). Indications of bone volume percentages achieved of other studies using hPDCs are shown in Table 6. Studies where bone volume percentage of a specific culture system is compared to the standard tissue flask system are interesting to compare with. Although the results of the multiplate bioreactor and the hollow fiber are slightly higher than the results of this work, they are not significantly different from their tissue flask control. This indicates that the variation between the systems is due to donor variability and not the cell culture system. The other bone percentage results from literature show that the cell-carrier combination of hPDC and NuOss is superior compared to Bio-Oss, Collagraft and Vitoss.

**TABLE 6:** Bone percentages after ectopic implantation of hPDCs on scaffolds in mice for 8 weeks. Data gathered from literature.

Reference	Vessel type	Scaffold	Bioreactor bone volume (%)	Tissue flask bone volume (%)
Lambrechts et al., 2016b	Multiplate	Nuoss	11.6 ± 3.1	12.8 ± 3.3
Lambrechts et al., 2016a	Hollow Fiber	Nuoss	10.3 ± 3.7	11.0 ± 3.8
Kerckhofs et al., 2016	Tissue flask	Chronos		13.13 ± 3.82
Roberts et al., 2011	Tissue flask	Nuoss		13.03 ± 3.57
Roberts et al., 2011	Tissue flask	Bio-Oss		5.13 ± 2.49
Roberts et al., 2011	Tissue flask	Collagraft		1.88 ± 1.35
Roberts et al., 2011	Tissue flask	Vitoss		3.21 ± 2.04

These results demonstrated that using spinner flasks with Star-Plus microcarriers has no significant difference in cell potency compared to tissue flask cultures. Besides comparing the results of this work with the standard used tissue flasks, other cell culture systems are also interesting to compare to. hPDCs have also been evaluated in a multiplate bioreactor (Xpansion, Pall life sciences) (Lambrechts et al., 2016b) and a hollow fiber bioreactor (Quantum, Terumo) (Lambrechts et al., 2016a), which both conclude that the cells retain their bone forming potency. The main differences and tradeoffs in cell culture systems are the price of the culture system, whether the system is re-usable or disposable, the efficiency of the downstream processes and how well the system can be automated to reduce variations and increase process efficiency.

## Conclusion

In conclusion, this work demonstrates that Star-Plus is a suitable microcarrier for the expansion of hPDCs in suspension culture. During this expansion process a xeno-free medium was used in 100 mL spinner flasks on different donors. Both *in vitro* assessment, but more importantly also *in vivo* assessments of the expanded hPDCs were carried out. The results of this work showed no significant difference in bone forming potential between the dynamic expansion and the standard tissue flask expansion. Therefore, this work presents a scalable production process of hPDCs using Star-Plus microcarriers in spinner flasks, where the cells maintain their bone forming potential *in vivo*. In this work we demonstrate that expansion efficiency, while safeguarding potency and cell functionality, can be obtained in a clinically relevant context.

## Data Availability Statement

The raw data supporting the conclusions of this article will be made available by the authors, without undue reservation.

## Ethics Statement

The animal study was reviewed and approved by KU Leuven Animal Ethics Committee.

## Author Contributions

KV and IP: conceptualization and investigation. KV: experimental work, formal analysis, data curation, writing–original draft preparation, and visualization. IP and J-MA: resources, supervision, and funding acquisition. KV, IP, and J-MA: writing–review and editing. J-MA: project administration. All authors have read and agreed to the published version of the manuscript.

## Conflict of Interest

The authors declare that the research was conducted in the absence of any commercial or financial relationships that could be construed as a potential conflict of interest.

## References

[B1] Durand C. Charbord P. (2015). *Stem cell biology and regenerative medicine.* Aalborg: River Publishers.

[B2] Allen M. R. Hock J. M. Burr D. B. (2004). Periosteum: Biology, regulation, and response to osteoporosis therapies. *Bone* 35 1003–1012. 10.1016/j.bone.2004.07.014 15542024

[B3] de Bari C. Dell’Accio F. Vanlauwe J. Eyckmans J. Khan I. M. Archer C. W. (2006). Mesenchymal multipotency of adult human periosteal cells demonstrated by single-cell lineage analysis. *Arthritis Rheum.* 54 1209–1221. 10.1002/art.21753 16575900

[B4] Duchamp de Lageneste O. Julien A. Abou-Khalil R. Frangi G. Carvalho C. Cagnard N. (2018). Periosteum contains skeletal stem cells with high bone regenerative potential controlled by Periostin. *Nat. Commun.* 9 773. 10.1038/s41467-018-03124-z PMC5823889 29472541

[B5] Nilsson Hall G. Mendes L. F. Gklava C. Geris L. Luyten F. P. Papantoniou I. (2020). Developmentally Engineered Callus Organoid Bioassemblies Exhibit Predictive In Vivo Long Bone Healing. *Adv. Sci.* 7 1902295. 10.1002/advs.201902295 31993293 PMC6974953

[B6] Slevin O. Ayeni O. R. Hinterwimmer S. Tischer T. Feucht M. J. Hirschmann M. T. (2016). The role of bone void fillers in medial opening wedge high tibial osteotomy: a systematic review. *Knee Surgery, Sport. Traumatol. Arthrosc.* 24 3584–3598. 10.1007/s00167-016-4297-5 27557796

[B7] Jung S. Panchalingam K. M. Wuerth R. D. Rosenberg L. Behie L. A. (2012). Large-scale production of human mesenchymal stem cells for clinical applications. *Biotechnol. Appl. Biochem.* 59 106–120. 10.1002/bab.1006 23586791

[B8] Beitzel K. McCarthy M. B. Cote M. P. Durant T. J. Chowaniec D. M. Solovyova O. (2013). Comparison of mesenchymal stem cells (osteoprogenitors) harvested from proximal humerus and distal femur during arthroscopic surgery. *Arthroscopy.* 29 301–308. 10.1016/j.arthro.2012.08.021 23290182

[B9] García-Fernández C. ópez-Fernández A. L. Borrós S. Lecina M. Vives J. (2020). Strategies for large-scale expansion of clinical-grade human multipotent mesenchymal stromal cells. *Biochem. Eng. J* 159 107601. 10.1016/j.bej.2020.107601

[B10] dos Santos F. F. Andrade P. Z. Da Silva C. L. Cabral J. M. S. (2013). Bioreactor design for clinical-grade expansion of stem cells. *Biotechnol. J.* 8 644–654. 10.1002/biot.201200373 23625834

[B11] Olsen T. R. Ng K. S. Lock L. T. Ahsan T. Rowley J. A. (2018). Peak MSC—Are We There Yet? *Front. Med.* 5:178. 10.3389/fmed.2018.00178 PMC6021509 29977893

[B12] Lambrechts T. Papantoniou I. Viazzi S. Bovy T. Schrooten J. Luyten F. P. (2016b). Evaluation of a monitored multiplate bioreactor for large-scale expansion of human periosteum derived stem cells for bone tissue engineering applications. *Biochem. Eng. J.* 108 58–68. 10.1016/j.bej.2015.07.015

[B13] Lambrechts T. Papantoniou I. Rice B. Schrooten J. Luyten F. P. Aerts J. M. (2016a). Large-scale progenitor cell expansion for multiple donors in a monitored hollow fibre bioreactor. *Cytotherapy* 18 1219–1233. 10.1016/j.jcyt.2016.05.013 27421744

[B14] Rojewski M. T. Fekete N. Baila S. Nguyen K. Fürst D. Antwiler D. (2013). GMP-compliant isolation and expansion of bone marrow-derived MSCs in the closed, automated device quantum cell expansion system. *Cell Transplant.* 22 1981–2000. 10.3727/096368912x657990 23107560

[B15] Jones M. Varella-Garcia M. Skokan M. Bryce S. Schowinsky J. Peters R. (2013). Genetic stability of bone marrow-derived human mesenchymal stromal cells in the Quantum System. *Cytotherapy* 15 1323–1339. 10.1016/j.jcyt.2013.05.024 23992670 PMC4116682

[B16] Hanley P. J. Mei Z. Durett A. G. Cabreira-Hansen Mda G. Klis M. Li W. (2014). Efficient manufacturing of therapeutic mesenchymal stromal cells with the use of the Quantum Cell Expansion System. *Cytotherapy* 16 1048–1058. 10.1016/j.jcyt.2014.01.417 24726657 PMC4087082

[B17] Nold P. Brendel C. Neubauer A. Bein G. Hackstein H. (2013). Good manufacturing practice-compliant animal-free expansion of human bone marrow derived mesenchymal stroma cells in a closed hollow-fiber-based bioreactor. *Biochem. Biophys. Res. Commun.* 430 325–330. 10.1016/j.bbrc.2012.11.001 23146633

[B18] Schnitzler A. C. Verma A. Kehoe D. E. Jing D. Murrell J. R. Der K. A. (2016). Bioprocessing of human mesenchymal stem/stromal cells for therapeutic use: Current technologies and challenges. *Biochem. Eng. J.* 108 3–13. 10.1016/j.bej.2015.08.014

[B19] de Bournonville S. Geris L. Kerckhofs G. (2021). Micro computed tomography with and without contrast enhancement for the characterization of microcarriers in dry and wet state. *Sci. Rep.* 11 2819. 10.1038/s41598-021-81998-8 PMC7854591 33531524

[B20] Loubière C. Sion C. De Isla N. Reppel L. Guedon E. Chevalot I. (2019). Impact of the type of microcarrier and agitation modes on the expansion performances of mesenchymal stem cells derived from umbilical cord. *Biotechnol. Prog.* 35 e2887. 10.1002/btpr.2887 31353825

[B21] Cimino M. Gonçalves R. M. Barrias C. C. Martins M. C. L. (2017). Xeno-free strategies for safe human mesenchymal stem/stromal cell expansion: Supplements and coatings. *Stem Cells Int.* 6597815 2017. 10.1155/2017/6597815 PMC5660800 29158740

[B22] Xia W. Li H. Wang Z. Xu R. Fu Y. Zhang X. (2011). Human platelet lysate supports ex vivo expansion and enhances osteogenic differentiation of human bone marrow-derived mesenchymal stem cells. *Cell Biol. Int.* 35 639–643. 10.1042/cbi20100361 21235529

[B23] Heathman T. R. J. Stolzing A. Fabian C. Rafiq Q. A. Coopman K. Nienow A. W. (2016). Scalability and process transfer of mesenchymal stromal cell production from monolayer to microcarrier culture using human platelet lysate. *Cytotherapy* 18 523–535. 10.1016/j.jcyt.2016.01.007 26971681

[B24] Oikonomopoulos A. van Deen WK Manansala A. R. Lacey P. N. Tomakili T. A. Ziman A. (2015). Optimization of human mesenchymal stem cell manufacturing: The effects of animal/xeno-free media. *Sci. Rep.* 5 16570. 10.1038/srep16570 PMC4643287 26564250

[B25] Gottipamula S. Sharma A. Krishnamurthy S. Sen Majumdar A. Seetharam R. N. (2012). Human platelet lysate is an alternative to fetal bovine serum for large-scale expansion of bone marrow-derived mesenchymal stromal cells. *Biotechnol. Lett.* 34 1367–1374. 10.1007/s10529-012-0893-8 22476583

[B26] Lohmann M. Walenda G. Hemeda H. Joussen S. Drescher W. Jockenhoevel S. (2012). Donor age of human platelet lysate affects proliferation and differentiation of mesenchymal stem cells. *PLoS One* 7:e37839. 10.1371/journal.pone.0037839 22662236 PMC3360602

[B27] Eyckmans J. Roberts S. J. Schrooten J. Luyten F. P. (2010). A clinically relevant model of osteoinduction: A process requiring calcium phosphate and BMP/Wnt signalling. *J. Cell. Mol. Med.* 14 1845–1856. 10.1111/j.1582-4934.2009.00807.x 19538476 PMC3829044

[B28] Gupta P. Hall G. N. Geris L. Luyten F. P. Papantoniou I. (2019). Human Platelet Lysate Improves Bone Forming Potential of Human Progenitor Cells Expanded in Microcarrier-Based Dynamic Culture. *Stem Cells Transl. Med.* 8 810–821. 10.1002/sctm.18-0216 31038850 PMC6646698

[B29] Rodrigues A. L. Rodrigues C. A. V. Gomes A. R. Vieira S. F. Badenes S. M. Diogo M. M. (2019). Dissolvable Microcarriers Allow Scalable Expansion And Harvesting Of Human Induced Pluripotent Stem Cells Under Xeno-Free Conditions. *Biotechnol. J.* 14 e1800461. 10.1002/biot.201800461 30320457

[B30] Nienow A. W. Rafiq Q. A. Coopman K. Hewitt C. J. (2014). A potentially scalable method for the harvesting of hMSCs from microcarriers. *Biochem. Eng. J.* 85 79–88. 10.1016/j.bej.2014.02.005

[B31] Chen Y. Sonnaert M. Roberts S. J. Luyten F. P. Schrooten J. (2012). Validation of a PicoGreen-Based DNA Quantification Integrated in an RNA Extraction Method for Two-Dimensional and Three-Dimensional Cell Cultures. *Tissue Eng. Part C Methods* 18 444–452. 10.1089/ten.tec.2011.0304 22195986

[B32] Hulspas R. (2010). Titration of fluorochrome-conjugated antibodies for labeling cell surface markers on live cells. *Curr. Protoc. Cytom* (Suppl. 54) 1–9. ^∗^vol, 10.1002/0471142956.cy0629s54 20938920

[B33] Schop D. van Dijkhuizen-Radersma R. Borgart E. Janssen F. W. Rozemuller H. Prins H. J. (2010). Expansion of human mesenchymal stromal cells on microcarriers: growth and metabolism. *J. Tissue Eng. Regen. Med.* 4 131–140. 10.1002/term.224 19842106

[B34] Frauenschuh S. Reichmann E. Ibold Y. Goetz P. M. Sittinger M. Ringe J. (2007). A microcarrier-based cultivation system for expansion of primary mesenchymal stem cells. *Biotechnol. Prog.* 23 187–193. 10.1021/bp060155w 17269687

[B35] Malda J. Kreijveld E. Temenoff J. S. Van Blitterswijk C. A. Riesle J. (2003). Expansion of human nasal chondrocytes on macroporous microcarriers enhances redifferentiation. *Biomaterials* 24 5153–5161. 10.1016/s0142-9612(03)00428-9 14568432

[B36] Betrachtungen T. (1968). Suspending of solid particles in liquid by agitators. *Chem. Eng. Sci.* 9 244–253. 10.1016/0009-2509(58)85031-9

[B37] Gupta P. Geris L. Luyten F. P. Papantoniou I. (2018). An Integrated Bioprocess for the Expansion and Chondrogenic Priming of Human Periosteum-Derived Progenitor Cells in Suspension Bioreactors. *Biotechnol. J.* 13 1700087. 10.1002/biot.201700087 28987025

[B38] Schop D. Janssen F. W. van Rijn LD Fernandes H. Bloem R. M. de Bruijn J. D. (2009). Growth, Metabolism, and Growth Inhibitors of Mesenchymal Stem Cells. *Tissue Eng Part A* 15 1877–1886. 19196147 10.1089/ten.tea.2008.0345

[B39] Lobner D. (2000). Comparison of the LDH and MTT assays for quantifying cell death: Validity for neuronal apoptosis? *J. Neurosci. Methods* 96 147–152. 10.1016/s0165-0270(99)00193-4 10720679

[B40] Kehoe D. Schnitzler A. Simler J. Dileo A. Ball A. (2012). Scale-up of human mesenchymal stem cells on microcarriers in suspension in a single-use bioreactor. *BioPharm Int.* 25 28–39.

[B41] Petry F. Smith J. R. Leber J. Salzig D. Czermak P. Weiss M. L. (2016). Manufacturing of human umbilical cord mesenchymal stromal cells on microcarriers in a dynamic system for clinical use. *Stem Cells Int* 4834616 2016. 10.1155/2016/4834616 PMC4761675 26977155

[B42] De Bari C. Dell’Accio F. Luyten F. P. (2001). Human periosteum-derived cells maintain phenotypic stability and chondrogenic potential throughout expansion regardless of donor age. *Arthritis Rheum.* 44 85–95. 10.1002/1529-0131(200101)44:1<85::aid-anr12>3.0.co;2-6 11212180

[B43] Rafiq Q. A. Coopman K. Nienow A. W. Hewitt C. J. (2016). Systematic microcarrier screening and agitated culture conditions improves human mesenchymal stem cell yield in bioreactors. *Biotechnol. J.* 11 473–486. 10.1002/biot.201400862 26632496 PMC4991290

[B44] Ferrari C. Balandras F. Guedon E. Olmos E. I (2012). Chevalot, and A. Marc, Limiting cell aggregation during mesenchymal stem cell expansion on microcarriers. *Biotechnol. Prog.* 28 780–787. 10.1002/btpr.1527 22374883

[B45] Jossen V. van den Bos C. Eibl R. Eibl D. (2018). Manufacturing human mesenchymal stem cells at clinical scale: process and regulatory challenges. *Appl. Microbiol. Biotechnol.* 102 3981–3994. 10.1007/s00253-018-8912-x 29564526 PMC5895685

[B46] Hewitt C. J. Lee K. Nienow A. W. Thomas R. J. Smith M. Thomas C. R. (2011). Expansion of human mesenchymal stem cells on microcarriers. *Biotechnol. Lett.* 33 2325–2335. 10.1007/s10529-011-0695-4 21769648

[B47] Dominici M. Le Blanc K. Mueller I. Slaper-Cortenbach I. Marini F. Krause D. (2006). Minimal criteria for defining multipotent mesenchymal stromal cells. The International Society for Cellular Therapy position statement. *Cytotherapy* 8 315–317. 10.1080/14653240600855905 16923606

[B48] dos Santos F. Andrade P. Z. Abecasis M. M. Gimble J. M. Chase L. G. Campbell A. M. (2011). Toward a Clinical-Grade Expansion of Mesenchymal Stem Cells from Human Sources: A Microcarrier-Based Culture System Under Xeno-Free Conditions. *Tissue Eng. Part C Methods* 17 1201–1210. 10.1089/ten.tec.2011.0255 21895491 PMC3226421

[B49] Potapova I. A. Brink P. R. Cohen I. S. Doronin S. V. (2008). Culturing of human mesenchymal stem cells as three-dimensional aggregates induces functional expression of CXCR4 that regulates adhesion to endothelial cells. *J. Biol. Chem.* 283 13100–13107. 10.1074/jbc.m800184200 18334485 PMC2442325

[B50] Frith J. E. Thomson B. Genever P. G. (2010). Dynamic three-dimensional culture methods enhance mesenchymal stem cell properties and increase therapeutic potential. *Tissue Eng. - Part C Methods* 16 735–749. 10.1089/ten.tec.2009.0432 19811095

[B51] Brown M. A. Wallace C. S. Anamelechi C. C. Clermont E. Reichert W. M. Truskey G. A. (2007). The use of mild trypsinization conditions in the detachment of endothelial cells to promote subsequent endothelialization on synthetic surfaces. *Biomaterials* 28 3928–3935. 10.1016/j.biomaterials.2007.05.009 17570483 PMC2025691

[B52] Ikeda T. Kawaguchi H. Kamekura S. Ogata N. Mori Y. Nakamura K. (2005). Distinct roles of Sox5, Sox6, and Sox9 in different stages of chondrogenic differentiation. *J. Bone Miner. Metab.* 23 337–340. 10.1007/s00774-005-0610-y 16133682

[B53] Ji W. Bolander J. Chai Y. C. Katagiri H. Marechal M. Luyten F. P. (2017). *Bone Morphogenetic Proteins: Systems Biology Regulators.* Cham: Springer.

[B54] Tucker D. Still K. Blom A. Hollander A. P. (2020). Over-Confluence of expanded bone marrow mesenchymal stem cells ameliorates their chondrogenic capacity in 3D cartilage tissue engineering. *bioRxiv.* 10.1101/2020.01.08.897645

[B55] Weiss H. E. Roberts S. J. Schrooten J. Luyten F. P. (2012). A Semi-Autonomous Model of Endochondral Ossification for Developmental Tissue Engineering. *Tissue Eng. Part A* 18 1334–1343. 10.1089/ten.tea.2011.0602 22394057

[B56] Kerckhofs G. Chai Y. C. Luyten F. P. Geris L. (2016). Combining microCT-based characterization with empirical modelling as a robust screening approach for the design of optimized CaP-containing scaffolds for progenitor cell-mediated bone formation. *Acta Biomater.* 35 330–340. 10.1016/j.actbio.2016.02.037 26925963

[B57] Roberts S. J. Geris L. Kerckhofs G. Desmet E. Schrooten J. Luyten F. P. (2011). The combined bone forming capacity of human periosteal derived cells and calcium phosphates. *Biomaterials* 32 4393–4405. 10.1016/j.biomaterials.2011.02.047 21421268

