# Supplementary figures and images for: Microcarrier Screening and Evaluation for Dynamic Expansion of Human Periosteum-Derived Progenitor Cells in a Xenogeneic Free Medium
Source: Front Bioeng Biotechnol. 2021 May 24;9:624890. doi: 10.3389/fbioe.2021.624890 (PMC8181150; doi:10.3389/fbioe.2021.624890)

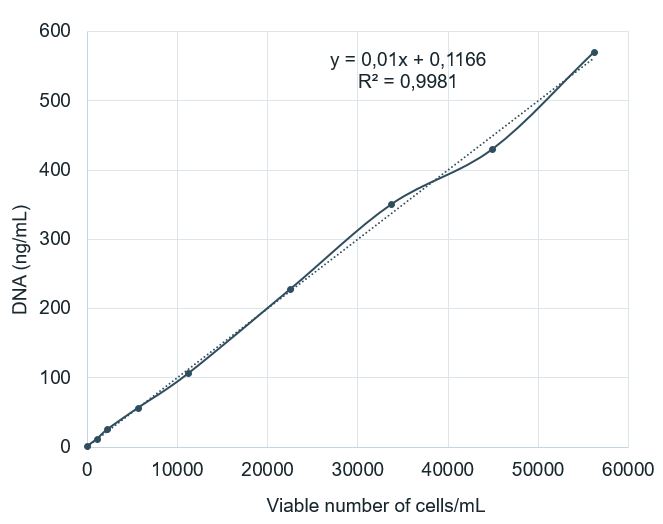

Supplement: Supplementary file 1 [file Image_1.JPEG]

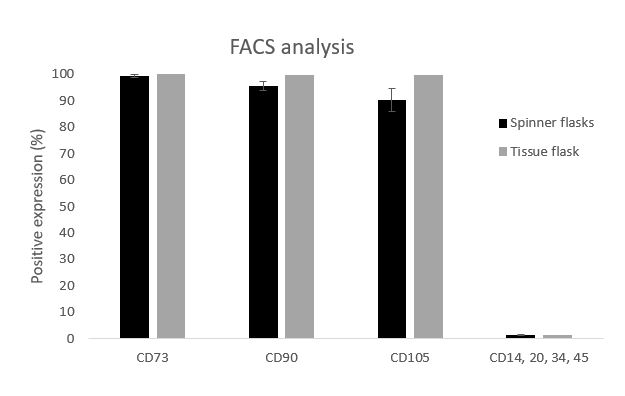

Supplement: Supplementary file 2 [file Image_2.JPEG]
